# Q fever in pregnant goats: humoral and cellular immune responses

**DOI:** 10.1186/1297-9716-44-67

**Published:** 2013-08-01

**Authors:** Hendrik IJ Roest, Jacob Post, Betty van Gelderen, Fred G van Zijderveld, Johanna MJ Rebel

**Affiliations:** 1 Department of Bacteriology and TSEs, Central Veterinary Institute, part of Wageningen University and Research Centre, Edelhertweg 15, Lelystad, PH, 8219, The Netherlands; 2 Department of Infection Biology, Central Veterinary Institute, part of Wageningen University and Research Centre, Edelhertweg 15, Lelystad, PH, 8219, the Netherlands

## Abstract

Q fever is a zoonosis caused by the intracellular bacterium *Coxiella burnetii.* Both humoral and cellular immunity are important in the host defence against intracellular bacteria. Little is known about the immune response to *C. burnetii* infections in domestic ruminants even though these species are the major source of Q fever in humans. To investigate the goat’s immune response we inoculated groups of pregnant goats via inhalation with a Dutch outbreak isolate of *C. burnetii*. All animals were successfully infected. Phase 1 and Phase 2 IgM- and IgG-specific antibodies were measured. Cellular immune responses were investigated by interferon-gamma, enzyme-linked immunosorbent spot test (IFN-γ Elispot), lymphocyte proliferation test (LPT) and systemic cytokines. After two weeks post inoculation (wpi), a strong anti-*C. burnetii* Phase 2 IgM and IgG antibody response was observed while the increase in IgM anti-Phase 1 antibodies was less pronounced. IgG anti-Phase 1 antibodies started to rise at 6 wpi. Cellular immune responses were observed after parturition. Our results demonstrated humoral and cellular immune responses to *C. burnetii* infection in pregnant goats. Cell-mediated immune responses did not differ enough to distinguish between *Coxiella*-infected and non-infected pregnant animals, whereas a strong-phase specific antibody response is detected after 2 wpi. This humoral immune response may be useful in the early detection of *C. burnetii*-infected pregnant goats.

## Introduction

Q fever is a zoonosis caused by *Coxiella burnetii*. *C. burnetii* has a worldwide distribution except for New Zealand [1]. The bacterium has a wide host range including humans, terrestrial and marine mammals, birds and reptiles [2,3]. The zoonotic impact of the disease has recently been underlined by the Dutch Q fever outbreak in which 4029 human cases were registered during the years 2007–2010 [4,5]. More than 40 000 people are assumed to be infected [6]. In this outbreak, *C. burnetii-*infected pregnant goats and sheep were the primary source of Q fever in humans [7,8]. During parturition these animals excrete high numbers of *C. burnetii* into the environment. Inhalation of *C. burnetii-*contaminated aerosols is the main route of infection in humans and can result in acute or chronic Q fever [9]. In the acute phase, humans suffer from a flu-like, self-limiting disease, atypical pneumonia or hepatitis. The chronic form of Q fever may lead to life-threatening endocarditis.

*C. burnetii* is a Gram-negative, intracellular bacterium. As in other Gram-negative bacteria (e.g. *Brucella* spp. and *Enterobacteriaceae* spp.), two major phenotypes (phases) of *C. burnetii* are recognised. Phase 1 of *C. burnetii* corresponds with the smooth phase of other Gram-negative bacteria and expresses full-length lipopolysaccharide (LPS) on its surface. Phase 2 corresponds with the rough phase of Gram-negative bacteria and lacks the O-antigenic region on its LPS [10]. Phase 1 is highly virulent and able to replicate in natural hosts, while Phase 2 *Coxiella* are considered avirulent and unable to replicate in immunocompetent animals [11,12]. The phase variation is interesting for the humoral immune response after *Coxiella* infection in mice, guinea pigs and humans. Following the inoculation of mice and guinea pigs with *C. burnetii* Phase 1, antibodies that recognise both Phase 1 and Phase 2 *C. burnetii* are generated [11,12]. In humans, the detection of phase-specific antibodies plays an important role in the diagnosis of acute and chronic Q fever [13]. This has not been investigated yet in goats, but is of importance specially in pregnant goats as these risk animals for human Q fever do not excrete *C. burnetii* during pregnancy and can therefore not be detected via the excretion of the bacterium [14]. Tools might be found that can help in the early diagnosis of Q fever in (pregnant) goats and can provide insights into the herd dynamics of Q fever infections, similar to those already anticipated in cattle herds [15].

The role of cellular immunity in the host defence against *C. burnetii* infections is not well established. In mice it is suggested that T cells are particularly important for the clearance of the bacterium after infection. Interferon-gamma (IFN-γ) and tumour necrosis factor-alpha (TNF-α) seem to be essential for the early control of *Coxiella* proliferation [12]. Furthermore, *in vitro* studies with human, peripheral blood, mononuclear cells indicate specific stimulation of T cells by human, monocyte-derived, dendritic cells (HMDCs) pulsed with *C. burnetii* outer membrane protein Com1 [16]. Recently the value of the interferon-gamma, enzyme-linked, immunosorbent spot test, a diagnostic tests based on cellular immunity, in the diagnosis of chronic Q fever in humans has also been shown [17]. In pre-vaccination screening of humans, a skin test is used to detect previously sensitised people so as to avoid adverse reactions after Q fever vaccination in these persons [18]. In cattle, a skin test with a diluted vaccine antigen has been suggested as a read out for a cellular response in an attempt to assess the duration of immunity after Q fever vaccination [19]. Cellular immune responses after *C. burnetii* infection of domestic ruminants have not been investigated, although this may provide potential tools to investigate the pathogenesis of *C. burnetii* infection in ruminants and to improve its diagnosis. In addition, the vaccine efficacy in already infected hosts can probably be improved if the cell-mediated immunity can be stimulated with a new generation of vaccines.

The goal of the present study was to investigate the humoral and cellular immune response in pregnant goats after inoculation with the Dutch outbreak strain of *C. burnetii*. The humoral immune response was measured by detecting anti-Phase 1 and anti-Phase 2 IgM- and IgG-specific antibodies. Cellular immune responses were measured with the interferon-gamma, enzyme-linked, immunosorbent spot test (IFN-γ Elispot) and the lymphocyte proliferation test (LPT) as well as by measuring systemic mRNA for different cytokines. Our results indicate a strong phase-specific IgM and IgG antibody response during early infection, whereas the cell- mediated immune response did not differ much between the *Coxiella*-infected pregnant goats and the non-infected animals.

## Materials and methods

### Inoculum

*C. burnetii* strain X09003262-001 was isolated from a placenta of a dairy goat that aborted on a farm during the Q fever outbreak in the Netherlands. This has previously been described [14]. In short, part of an immunohistochemically confirmed *C. burnetii*-positive placenta was crushed and filtered before inoculation with a culture of Buffalo Green Monkey (BGM) cells kept in culture medium without antibiotics (EMEM with 10% bovine serum albumin, 1% NEAA, 1% glutamax). The inoculated cells were incubated for 14 days at 37°C in a closed flask and culture medium was refreshed twice a week. The cell culture was negative for *Chlamydia abortus, Simkania negevensis* and mycoplasma. A large batch of strain X09003262-001 was prepared and the mouse-infective dose (MID) of the batch was determined. Prior to inoculation, the inoculum was adjusted to the required MID by dilution with culture medium. The strain was genotyped as CbNL01, the predominant *C. burnetii* genotype in the Dutch Q fever outbreak. To ensure inoculation of Phase 1 bacteria, cell culture passage 2 of the field isolate was used. In the inoculum, no Phase 2 *C. burnetii* were detected with an immunofluorescence test set up with the serum of a goat with a high anti-Phase 2 antibody titre but no Phase 1 titre. All experiments were approved by the Animal Experiment Commission of the Central Veterinary Institute, part of Wageningen UR, in accordance with Dutch regulations on animal experimentation.

### Animal experiment

The experimental set up has previously been described (Experiment III, [14]). Sixteen healthy, pregnant, serologically Q fever negative, Alpine yearling goats were purchased from INRA (Institut National de la Recherche Agronomique, Domaine de Galle), France. Pregnancy and the duration of pregnancy were confirmed using ultrasound. All goats tested serologically negative for antibodies against *C. burnetii* and *Chlamydia abortus* on the day of arrival. Six negative control goats were housed in animal biosafety level (aBSL) 2 facilities. Two groups of 5 goats (Group A and B) were separately housed in aBSL3 facilities for inoculation with *C. burnetii.* On day 76 of pregnancy, 10 goats were intranasally inoculated with 1 mL containing 10^6^ MID *C. burnetii* while the six negative control animals were intranasally inoculated with 1 mL of culture medium. General health was monitored by daily clinical inspection of behaviour, appetite and consistency of the faeces. All goats were kept alive until the end of the experiment at 13 weeks post inoculation (wpi) (negative controls and Group A) and 14 wpi (Group B). Weak-born kids were euthanised when necessary for ethical reasons and liveborn kids were kept together with their does until the end of the experiment.

During the experiment goats did not show any clinical signs of disease except for abortion. Three of the ten *Coxiella*-inoculated goats aborted (one single kid and two twins) at 7, 8 and 10 wpi respectively. One goat delivered one stillborn kid and one liveborn weak kid at 10 wpi. Two goats delivered one stillborn and one liveborn healthy kid each at 9 wpi; one goat delivered two weak kids at 9 wpi and three goats delivered healthy kids, all singles at 9 wpi. The six control goats delivered healthy liveborn kids at 10 and 11 wpi, which was equal to 149 to 157 days of gestation. *C. burnetii* was detected in the placentas of all ten *Coxiella*-inoculated goats by immunohistochemistry and by PCR in the vaginal mucus just after parturition. These results indicate that all *Coxiella*-inoculated goats were successfully infected with *C. burnetii*.

### Sampling

Jugular blood was sampled from each of the goats weekly from 0 wpi just before the inoculation until 13 wpi, except that at 4 wpi goats were not sampled and at 13 wpi the negative control goats were not sampled for antibody detection for logistical reasons. When results from samples were related to parturition, parturition was set at time point 0; results from samples taken in the week before parturition were indicated as week −1 and results from samples taken in the week after parturition were indicated as week 1. Blood was collected in coagulation tubes for antibody detection, in anticoagulation tubes (EDTA) for cell-mediated immunity and in PAXgene® Blood RNA tubes to preserve cytokine’s mRNA.

### Detection of phase 1 and phase 2 IgM and IgG antibodies

*C. burnetii* Phase 1 and Phase 2 IgM- and IgG-specific antibodies were detected in an ELISA format. *C. burnetii* Phase 1 and Phase 2 ELISA-specific plates were purchased from Virion/Serion (Serion ELISA classic *Coxiella burnetii* Phase 1 and Phase 2, Germany). Optimal serum and conjugate dilutions were determined in advance and positive and negative controls were selected (data not shown). Plates were incubated with 100 μL 1:160 diluted serum in phosphate buffered saline (PBS), pH 7.2 with 0.5 ml 10% (v/v) tween 80 (PBS-Tw) for 1 h at 37°C. After incubation, plates were washed automatically (Schleicher, Germany), 6 times with 1400 μL of 0.5 ‰ Tween 20 in water and incubated for 1 h at 37°C with 100 μL of diluted alkaline phosphatase-conjugated antibodies. For the detection of IgM antibodies rabbit anti-goat IgM (Bioconnect, the Netherlands) antibodies were used, 1:1000 diluted in PBS-Tw and 0.5 M NaCl for the detection of Phase 1 antibodies or 1:5000 diluted for the detection of Phase 2 antibodies. For the detection of IgG antibodies rabbit F(ab’)2 anti-goat IgG (H/L) (Bioconnect, the Netherlands) were used, 1:2000 diluted for the detection of Phase 1 antibodies or 1:4000 diluted for the detection of Phase 2 antibodies. After incubation with the conjugate, plates were washed as described above and 100 μL of para-nitrophenylphosphate substrate (Virion/Serion, Germany) per well was added and the reaction was stopped after 30 min at 37°C with 100 μL of 1.2 N sodium hydroxide (Virion/Serion, Germany). The optical density (OD) was measured at 405 nm (EL 808 Ultra microplate reader, Bio-tek instruments, USA). On each plate the same negative and positive control serum was tested in duplicate per phase/Ig combination. Results of the serum were given related to the average positive control OD, both corrected for the average negative control OD.

### Interferon-gamma, enzyme-linked, immunosorbent spot test (IFN-γ Elispot)

Peripheral blood mononuclear cells (PBMCs) were isolated from EDTA blood by Ficoll-Hypaque (Amersham Biosciences, Sweden) density gradient centrifugation. Interferon-gamma, enzyme-linked immunosorbent spot test (IFN-γ Elispot) assay was performed using 5 × 10^5^ PBMCs per well (MSIPS4W10 plates, Millipore, USA) from each goat. Detection of the T cell-produced IFN-γ was performed with the Elispot kit for Bovine/Ovine/Equine IFN-γ (MabTech, Nacka Strand, Sweden). We evaluated the kit for caprine IFN-γ and found it suitable for use (data not shown). *C. burnetii* T-cell responses were examined after stimulation with culture medium (negative control), *C. burnetii* strain Nine Mile Phase 1 and Phase 2 (Virion/Serion 1227, 1:5000 diluted in culture medium) or ConA (positive control). IFN-γ spot-forming cells were counted using an ImmunoSpot analyzer (CTL, USA). We optimised counting parameters to precisely and accurately count all Elispot plates. The average results of all goats in the group are given as relative results of the positive control after correction for the medium control.

### Lymphocyte proliferation test (LPT)

PBMCs suspended in medium (negative control), *C. burnetii* strain Nine Mile Phase 1 and Phase 2 (Virion/Serion 1227, 1:5000 diluted in culture medium) or ConA (positive control) were added to triplicate wells. Plates were incubated at 37°C for 72 h. For the last 18 h of incubation, alamar blue (Invitrogen, USA) was added. Supernatant was harvested and absorbance was measured at 570 nm/600 nm. Results are given as average results per group. Individual results were calculated as relative induction of the average difference of the OD at 600 nm and 570 nm of the sample in duplicate compared to the medium control.

### Cytokine mRNA induction

Blood specimens (2.5 mL) collected in PAXgene® tubes were incubated at room temperature for 4 h for RNA stabilisation and then stored at −80°C. RNA was extracted from whole blood using the manufacturer’s guidelines. In brief, samples were removed from −80°C and incubated over night at 4°C to ensure complete lysis of the blood cells. Then tubes were centrifuged for 10 min at 4000 *g*, the supernatant was discarded and 5 mL of RNase-free water was added to the pellet. The pellet was resuspended. Washing was repeated and the pellet was finally resuspended in 1 mL trizol (Invitrogen, USA). Subsequently, a phase separation with chloroform was performed and RNA was precipitated using 2-propanol. Additional purification was performed with the DNA-free kit (Ambion, USA). The quality and integrity of the RNA samples were analysed using the Agilent Bioanalyzer (lab on chip, Agilent Technologies, USA). For the quantification of cytokine mRNA, cDNA was made using random hexamer primers and reverse transcriptase. Forward and reverse primers were selected to detect the cDNA of TNF-α, IL-1β, IFN-α, IFN-γ, IL-2 and IL-10 (Table 1). PCR was performed using Syber Green PCR Master Mix (Applied Biosystems, USA) in an ABI 7500 Real-Time PCR system (PE Applied Biosystems, USA). Results were quantified and normalised compared to the amount of succinate dehydrogenase complex subunit A gene (SDHA) of the same sample. SDHA was selected as reference gene on basis of the geNORM Bos Taurus housekeeping gene selection kit (Primerdesign, UK) as the best performing housekeeping gene compared to the other housekeeping genes included in the kit. For the quantification, a standard curve of the plasmid with the insert of the cytokine of interest constructed in pGEM-T easy (Promega, the Netherlands) was used. For the determination of the amount of the reference gene per sample, a standard curve of mRNA SDHA in water was used. For negative controls, RNA samples without reverse transcriptase in the reaction mixture were used.

**Table 1:** Sequences of the forward en reverse primers used to detect the DNA transcripts of the mRNA of goat’s TNF-α, IL-1β, IFN-α, IFN-γ, IL-2 and IL-10

**Gene of interest**	**NCBI code**	**Primer**	**Sequence**
TNF-α	X14828	Forward	CCTTGAGAAGATCTCACCTA
		Reverse	CAAACATAAACAGAGGGAGT
IL-1β	DQ837160	Forward	TACCTGTCTTGTGTGAAAAA
		Reverse	CAAATTCAACTGTGTTCTTG
IFN-α	FJ959074	Forward	GAGGAAATACTTCCACAGAG
		Reverse	ATGACTTCTGCTCTGACAAC
IFN-γ	EF375708	Forward	GAAATTTTGAAGAATTGGAA
		Reverse	AATGACCTGGTTATCTTTGA
IL-2	AF535145	Forward	GATGTCTAGAAGCAAGGGTA
		Reverse	ACATCCAAATGAGTTCTGTT
IL-10	DQ837159	Forward	GGCAAAGTGAAGACTTTCT
		Reverse	ACTGGATCATTTCTGACAAG

*NCBI* National Center for Biotechnology Information.

### Statistical analyses

A two-way analysis of variance (ANOVA) with infection status (i.e. infected with *C. burnetii* and non-infected goats) and sample week as independent variables and the results of the used tests (i.e. phase specific ELISAs, IFN-γ Elispot, LPT and the different cytokines) as dependent variable was used to compare the difference between de parameters of the two groups of animals (IBM SPSS Statistics 19). Homogeneity of variances was preliminary tested using Levene’s test. *P* values ≤ 0.05 correspond with a confidence interval of 95%, marked as *; *P* ≤ 0.01 correspond with a confidence interval of 99%, marked as **.

## Results

### Humoral immune response

**Figure 1:**
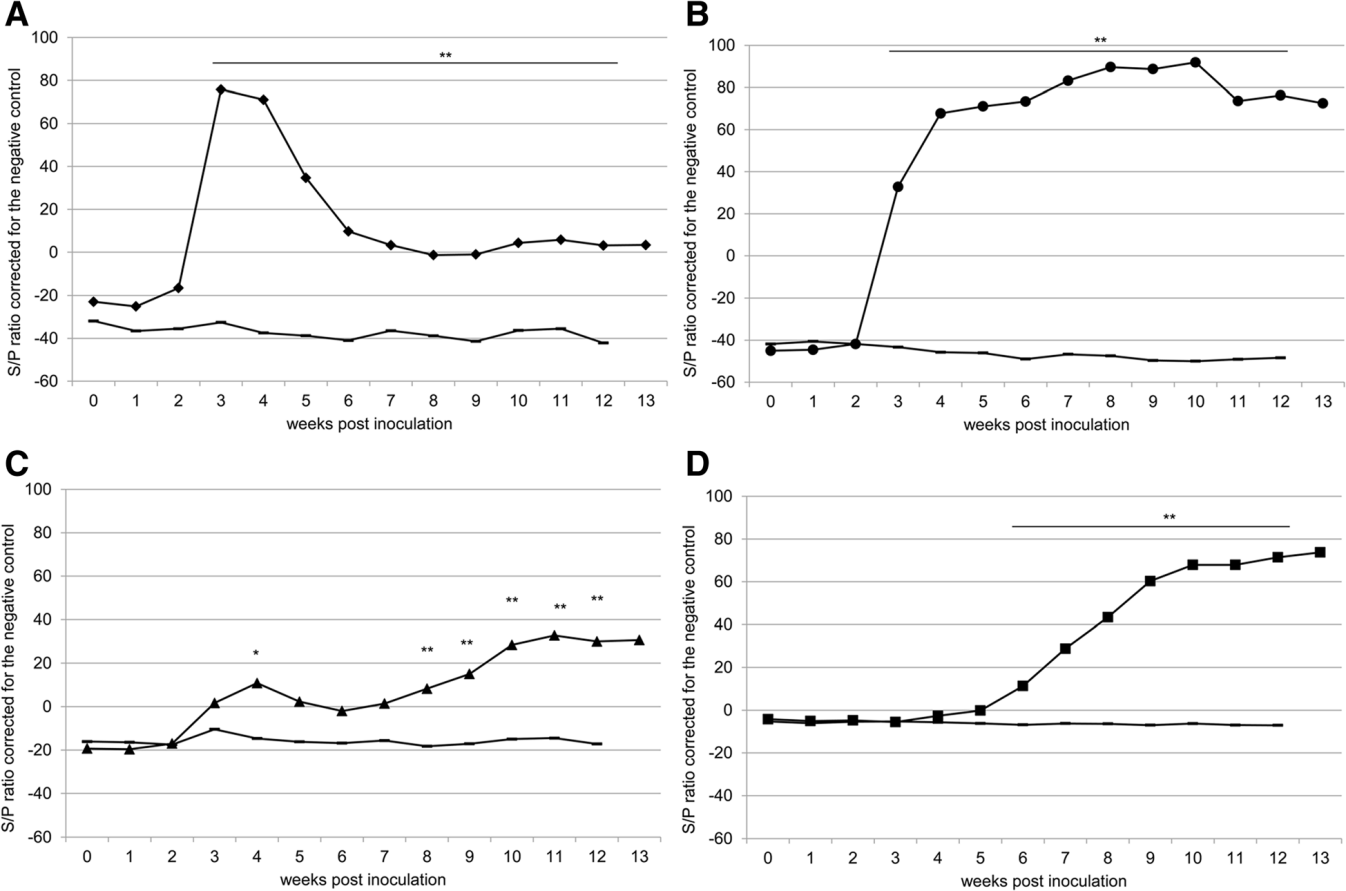
**Results of the *C. burnetii *phase 1 and phase 2 IgM and IgG specific ELISA.** Average antibody titres expressed as S/P ratio corrected for the negative control for IgM Phase 2 **(**IgMph2, ♦, **A)** and Phase 1 **(**IgMph1, ▲, **C)** and IgG Phase 2 **(**IgGph2, ●, **B)** and Phase 1 **(**IgGph1, ■, **D)** as measured with an IgM- and IgG- specific conjugate in *C. burnetii* phase-specific ELISA. (−): negative control goats *: *P* ≤ 0.05; **: *P* ≤ 0.01.

Ten pregnant goats were intranasally inoculated with a Dutch *C. burnetii* outbreak strain and jugular blood was sampled weekly. The serum of each goat was tested for IgM and IgG *C. burnetii*-specific antibodies using *C. burnetii* Phase 1- and Phase 2-specific ELISAs. Average IgM antibody levels against Phase 2 antigen (IgMph2) rised significantly from three weeks post-inoculation (wpi) onwards; after 3 wpi the antibody titre decreased and stabilised until the end of the experiment (Figure 1a). Average anti-*C. burnetii* Phase 2 IgG antibody levels (IgGph2) rapidly increased between 2 wpi and 4 wpi and then at a slower rate till 10 wpi (Figure 1b). Average anti-*C. burnetii* Phase 1 IgM antibody levels (IgMph1) started to rise from 3 wpi onwards and a significant difference between the infected and non-infected group was present at 4 wpi and from 8 wpi till the end of the experiment (Figure 1c). Anti-*C. burnetii* Phase 1 IgG antibody levels (IgGph1) started to rise at 6 wpi; at 9 wpi the average titre stabilised until the end of the experiment at 12 wpi (Figure 1d). IgMph2 and IgGph2 levels were not influenced by parturition. The second rise of IgMph1 started 1 week before parturition. IgGph1 started to rise 4 weeks before parturition. The data indicate a strong IgMph2 and IgGph2 response starting after two weeks after inoculation. The IgMph1 and IgGph1 response started later after inoculation.

### Cell-mediated immune response

**Figure 2:**
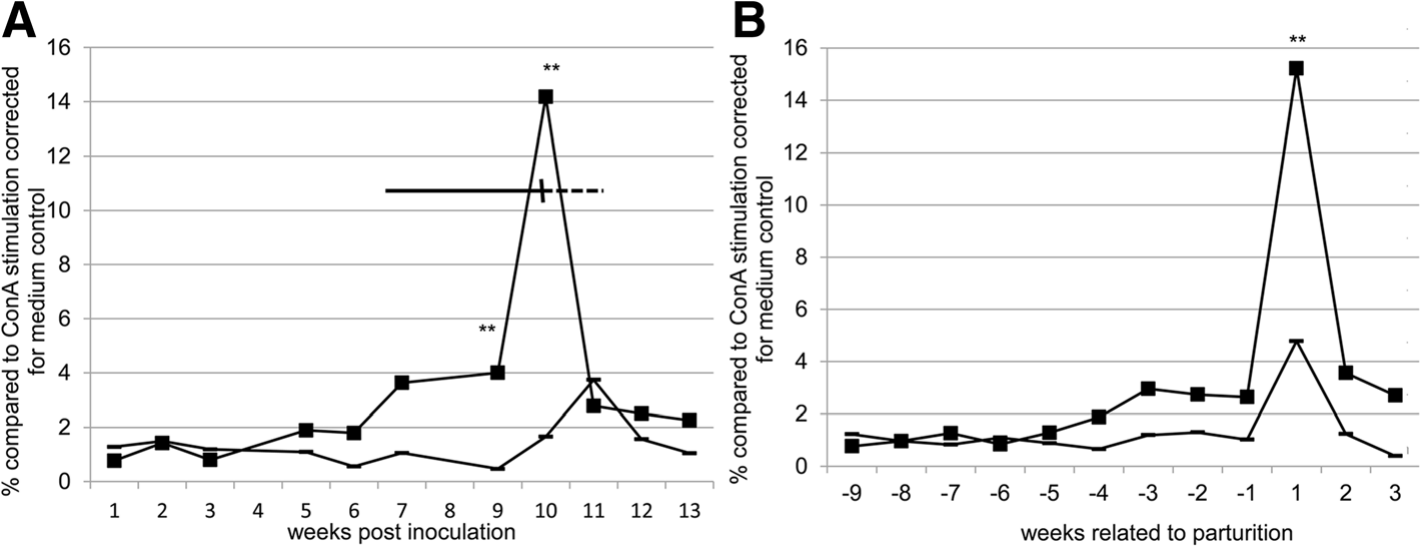
**IFN-y Elispot results, related to inoculation and to parturition.** Average data for PBMCs stimulated with Phase 1 and Phase 2 antigen. Data were corrected for medium-incubated cells and expressed as a percentage of ConA-stimulated cells. **A**: data were related to inoculation. Solid bar: delivery period of the *Coxiella*-inoculated goats; dotted bar: delivery period of the control goats. **B**: data related to delivery. (■) *Coxiella*-inoculated goats; (**−**) negative control goats; *: *P* ≤ 0.05; **: *P* ≤ 0.01.

To investigate the cell-mediated immune response, IFN-γ Elispot and the LPT were performed on cells isolated from the jugular blood samples. In these tests *C. burnetii* Phase 1 and Phase 2 antigens were used as stimulus. In the first four weeks after inoculation, no significant difference between the *Coxiella*-inoculated goats and the control goats was observed in the IFN-γ production by peripheral blood mononuclear cells (PBMCs) after stimulation with *C. burnetii* Phase 1 and Phase 2 antigen (Figure 2a).

**Figure 3:**
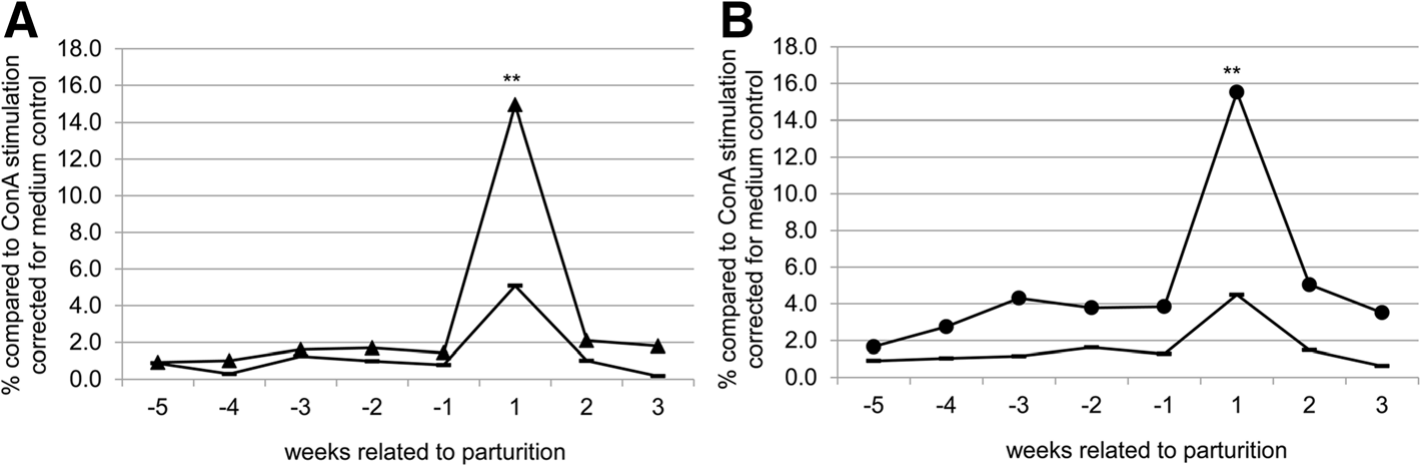
**IFN-y Elispot results, per *C. burnetii *phase 1 and phase 2 stimulus, related to parturition.** Results of the PBMC of control goats and *Coxiella*-inoculated goats after stimulation with *C. burnetii* Phase 1 or Phase 2 antigen. Data were corrected for medium-incubated cells and expressed as percentage of ConA-stimulated cells, the X-axis is in weeks related to parturition (time point 0). **A**: results after stimulation with *C. burnetii* Phase 1 antigen, (▲: *C. burnetii*-inoculated goats; **−**: control goats). **B**: results after stimulation with *C. burnetii* Phase 2 antigen (●: *C. burnetii*-inoculated goats; **−**: control goats); *: *P* ≤ 0.05; **: *P* ≤ 0.01.

**Figure 4:**
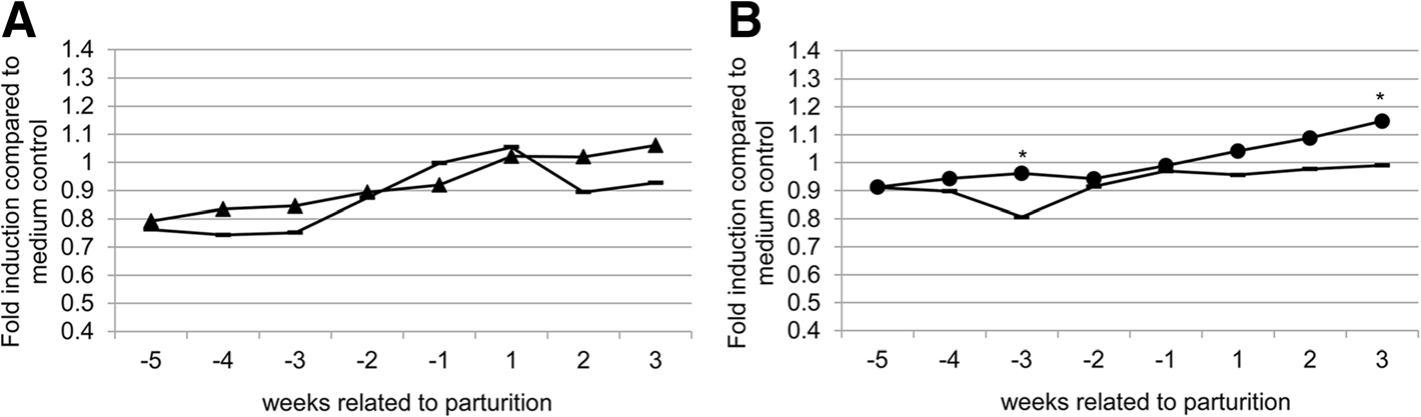
**LPT results, per *C. burnetii *phase 1 and phase 2 stimulus, related to parturition.** Lymphocyte proliferation test results of the control goats and *Coxiella*-inoculated goats. Data are expressed as average results of the group in fold induction compared to medium-incubated cells. The X-axis indicates weeks related to parturition (time point 0). **A**: results after stimulation with *C. burnetii* Phase 1 (▲: *C. burnetii*-inoculated goats; −: control goats). **B**: results after stimulation with *C. burnetii* Phase 2 antigen (●: *C. burnetii*-inoculated goats; −: control goats); *: *P* ≤ 0.05; **: *P* ≤ 0.01.

More detailed analysis of the IFN-γ Elispot results revealed a strong increase of the IFN-γ producing PBMCs around the date of parturition for both *Coxiella*-inoculated goats and control goats. Upon correcting for the date of parturition, the increase in IFN-y production in both *Coxiella*-inoculated and control goats corresponded to the time of parturition (Figure 2b), suggesting that parturition influences IFN-y production in goats. In the further analysis of the IFN-γ Elispot and LPT results, data were corrected for parturition. Stimulation with both Phase 1 and Phase 2 antigen resulted in a significant increase of the production of IFN-γ one week after parturition (Figure 3a and b). LPT results revealed an increase in proliferation ability of the PBMCs only after parturition in the *Coxiella*-inoculated goats (Figure 4a and b). The increase in proliferation ability after stimulation with *C. burnetii* Phase 2 antigen was stronger compared to stimulation with Phase 1 antigen (Figure 4b). Taken together, no clear cell-mediated immune response could be detected in the first weeks after infection. Only after parturition a significant increase in cell-mediated immune response was measured.

### Systemic cytokine mRNA responses

**Figure 5:**
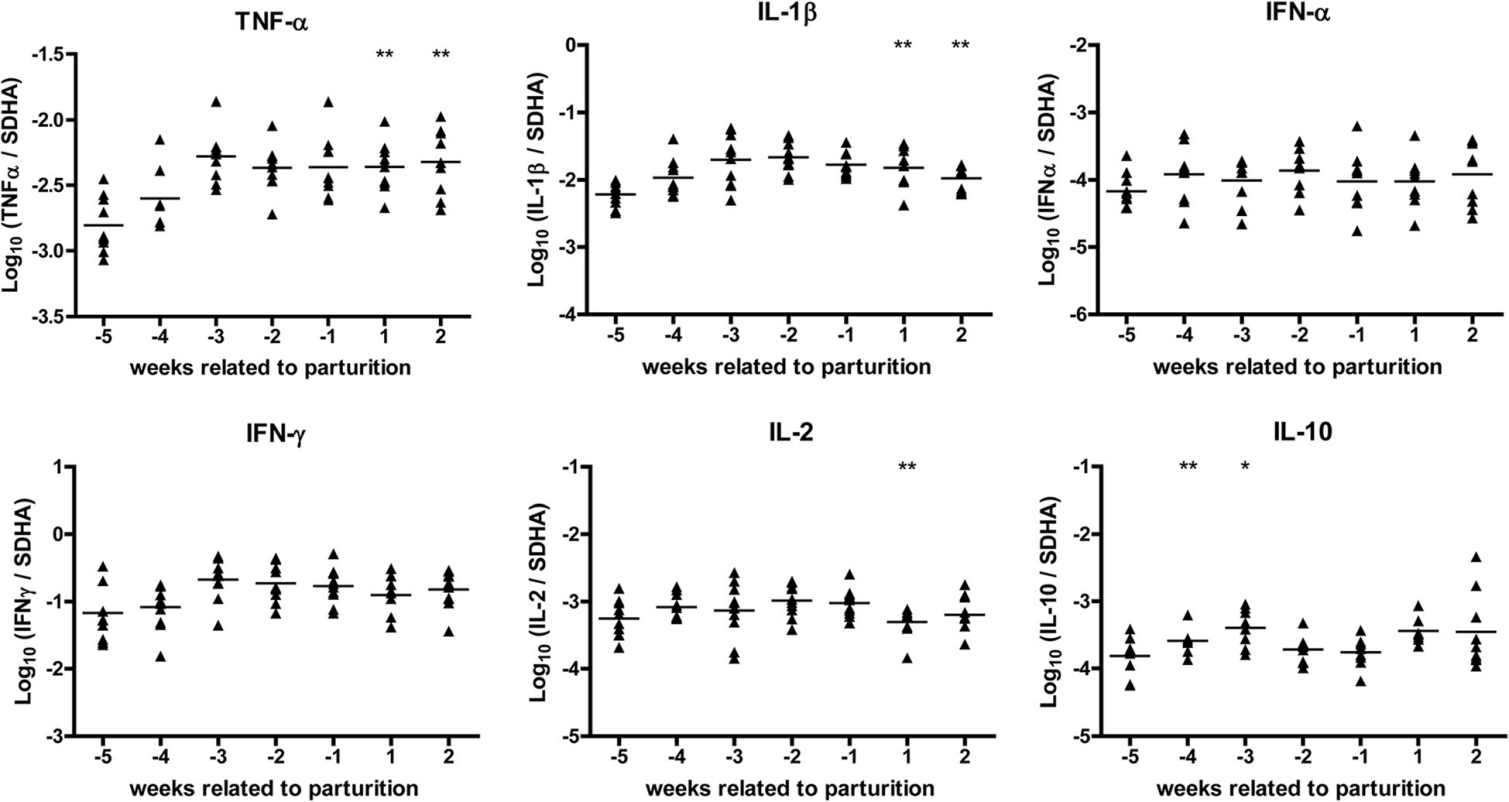
**Results of the systemic cytokine mRNA response detected by qPCR.** The results show cytokine mRNA levels measured by qPCR in the blood of *Coxiella*-inoculated goats normalised to SDHA in the period around parturition. The presence of cytokine mRNA in peripheral blood was examined in *Coxiella*-inoculated goats and in control goats every week post inoculation but no differences were found until the displayed period. Triangles represent the data of individual *Coxiella*-inoculated goats. The horizontal line represents the mean of the group at that time point. Significant elevation of the systemic mRNA levels between *Coxiella*-inoculated and control goats is indicated as * for *P* ≤ 0.05 and ** for *P* ≤ 0.01.

Additional information about the cellular and humoral immune response was obtained via weekly measurement of systemic mRNA levels of the regulation of the pro-inflammatory cytokines TNF-α and IL-1β and the regulatory cytokines IL-2 and IL-10. IFN-α and IFN-γ mRNA regulation was measured likewise. Within 4 weeks after inoculation no differential effect on cytokine mRNA levels was measured between the *Coxiella*-inoculated group compared to the control group (data not shown). Systemic cytokine responses related to parturition are presented in Figure 5. The pro-inflammatory cytokines TNF-α and IL-1β mRNA were up regulated after parturition compared to the non-infected goats. No effect could be observed on IFN-α- and IFN-γ mRNA levels. For the regulatory cytokines IL-2 and IL-10 mRNA, IL-2 was down regulated one week after parturition while IL-10 was up regulated four and three weeks before parturition (Figure 5). Results indicate a significant systematic up regulation of TNF-α, IL-1β and IL-2 after parturition and a up regulation of IL-10 before parturition in the *Coxiella*-inoculated goats compared to the control goats.

## Discussion

The goal of this experimental longitudinal study was to investigate the humoral and cellular immune responses in pregnant goats infected via a natural infection route with a Dutch outbreak isolate of *C. burnetii*. The immune response after *C. burnetii* inoculation via inhalation in domestic ruminants had not been studied yet. This inoculation route was successful in infecting goats and resulted in *C. burnetii*-infected placentas [14]. Up until now only subcutaneous inoculations had been used to study humoral immune responses [20,21]. However, the inoculation route might be important for studying naturally occurring immune responses.

Our results indicate a strong IgM anti-Phase 2 and IgG anti-Phase 2 humoral response, which starts to rise between 2 to 3 weeks post inoculation. This time period between inoculation and the first antibody response is shorter than reported for non-phase-specific antibodies in goats after subcutaneous inoculation [20,21], but is slightly longer than reported for humans and mice [22,23]. For goats, the difference with previous studies may be due to the use of different inoculation routes. Inoculation via inhalation can generate a mucosal immune response in the lungs; in humans, the lungs have the ability to respond quickly to some pathogens, i.e. *Streptococcus pneumoniae*. In the lungs, residential antigen-specific memory B cells are present but it is also reported that infections in the lung generate a *de novo* local and systemic antibody response [24]. In goats it is not known whether *C. burnetii* can induce such a response in the lung but the fast systemic antibody response could be a result of this.

The initial humoral immune response consisted of the generation of *C. burnetii* Phase 2-specific antibodies, whereas antibodies against *C. burnetii* Phase 1 arose at a later stage. Although in line with previously published work in mice, guinea pigs and humans [22,23,25], it is not clear why an anti-Phase 2 response is generated in advance of an anti-Phase 1 response, as Phase 1 *Coxiella* bacteria were inoculated. One possible explanation is that besides Phase 1 bacteria, Phase 2 bacteria were inoculated as well. Phase 2 bacteria are efficiently internalised into phagosomes [9,26]. This results in an effective killing of the Phase 2 bacteria that gives rise to a humoral response against Phase 2 *C. burnetii*. Alternatively, it can be assumed that both LPS and surface protein antigens of Phase 1 *C. burnetii* are recognised by the immune system but that surface proteins, which Phase 1 and Phase 2 *C. burnetii* have in common [27], give an earlier and stronger humoral immune response compared to the LPS antigen. This results first in an anti-Phase 2 response to the surface antigens, followed by an anti-Phase 1 response against LPS. As we were not able to detect Phase 2 *C. burnetii* in our inoculum, we assume that both surface proteins and LPS are detected by the immune system. Therefore the initial anti-Phase 2 response may be due to an earlier and stronger response to surface proteins than to LPS.

Surprisingly both anti-Phase 2 IgM and IgG titres started to rise at almost the same time. This was not expected, as IgM is generally the first immunoglobulin class to be produced in a humoral immune response because IgM can be expressed without class switching. During human *Coxiella* infection, IgM anti-Phase 2 antibody titres also start to rise first followed by a IgG anti-Phase 2 response [22]. The phase-specific and antibody-subclass-specific humoral immune response might help in the detection of early or more prolonged *C. burnetii* infections in goats. As pregnant goats do not excrete the bacterium [14] the detection of anti-Phase 2 IgM without anti-Phase 1 IgG will indicate an early infection. Whether phase-specific and antibody-subclass-specific antibody titres can predict or indicate chronic infections in goats should be investigated in further research, as the study period in the present study was only 13 weeks.

The cell-mediated immune response during the first weeks after inoculation was minimal, as indicated by the results of the IFN-γ Elispot and the apparent absence of a systemic cytokine mRNA responses. This might indicate that the PBMCs have not been in contact with *C. burnetii.* This corresponds with our earlier results, which showed that *Coxiella* bacteria were not detectable in the blood after inoculation despite the infection of the trophoblasts of the placenta between 2 and 4 weeks after inoculation [14]. Studies in non-pregnant mice indicate that IFN-γ has a role in the early control of *C. burnetii* proliferation [12]. This is probably not true in pregnant goats because we were not able to detect an increase in IFN-γ producing cells in the weeks after inoculation. Therefore IFN-γ probably does not play a role in preventing *C. burnetii* replication in the early stages of infection in pregnant goats.

Cell-mediated immune responses were first detected in the first week after parturition. At the time of parturition, the *C. burnetii* DNA load in the tissues is maximal as previously shown [14], resulting in exposure to the systemic immune system. This exposure coincides with an up regulation of the pro-inflammatory cytokines TNF-α and IL-1β. This probably caused the strong increase in the total production of IFN-γ by PBMCs at one week post parturition and the increase of the proliferation ability of PBMCs. However, parturition in non-infected goats also coincided with an increase in IFN-γ producing PBMCs as measured in the control goats. This could be an effect of the release of suppression of the cell-mediated immune response after parturition. During pregnancy progesterone levels are high and decrease shortly before parturition [28]. In humans it is suggested that high progesterone levels are associated with a Th-2 type immunity, resulting in an increased humoral immune response and a decreased cell-mediated immune response [29]. In pregnant goats we also assume a down regulation of the cell-mediated immune response due to progesterone during pregnancy. If the suppression is alleviated after parturition, a strong increase in IFN-γ producing cells can be expected. Overall, we assume that two mechanisms probably influence the cell-mediated immune response in *Coxiella*-infected pregnant goats, i.e. a stimulation by the release of *Coxiella* antigens and a suppression of cell-mediated immunity due to progesterone, with the overall effect that in infected goats no cell-mediated immune response was found during pregnancy.

IL-10 was up regulated towards the end of pregnancy in the *Coxiella*-inoculated goats. In mice as well as in humans persistent *Coxiella* presence seems to be IL-10 dependent [9,30] and humans suffering from chronic Q fever excrete high levels of IL-10 [9]. It can be hypothised that *Coxiella*-infected pregnant goats may react similar as persistently infected mice and humans and reach a chronic infection status which is terminated by the induction of parturition. However, little is known about persistence of *Coxiella* infections in goats and how to detect these animals. In our earlier experiments on the pathogenesis of Q fever in pregnant goats we were not able to detect *C. burnetii* DNA at 81 days after parturition [14] although others were able to detect *C. burnetii* DNA in the genital tract of non-pregnant goats [31] and field observations may indicate long lasting shedding of *C. burnetii* in previously infected non-pregnant dairy goats [32]. To elucidate this further research is needed.

The results of our study may have several implications for diagnostic applications of immunological tests for Q fever in pregnant animals. Cell-mediated immune responses did not differ enough to distinguish between *Coxiella*-infected and non-infected pregnant goats. IFN-γ Elispot, LPT and an additional performed IFN-γ ELISA (data not shown, as results were over all even less discriminative) have no additional value in the diagnosis of Q fever in pregnant goats. The strong humoral response, however, is useful in the early detection of infected pregnant goats, because these goats cannot be diagnosed by the detection of *C. burnetii*[14]. The phase-specific IgM and IgG response might be useful in understanding the dynamics of Q fever in a herd, as animals in different stages of infection can be followed.

## Competing interests

The authors declare that they have no competing interests.

## Authors’ contributions

Conceived and designed the experiments: HIJR, JP, EvG, FGvZ, JMJR. Performed the experiments: HIJR, JP, EvG. Analyzed the data: HIJR, JP, EvG, JMJR. Wrote the paper: HIJR, JP, EvG, FGvZ, JMJR. All authors read and approved the final manuscript.
